# Corrigendum to “Antibiotic bone cement accelerates diabetic foot wound healing: Elucidating the role of ROCK1 protein expression”

**DOI:** 10.1111/iwj.14889

**Published:** 2024-06-02

Yang C, Wang D. Antibiotic bone cement accelerates diabetic foot wound healing: elucidating the role of ROCK1 protein expression. Int Wound J. 2024;21(4):e14590. doi:10.1111/iwj.14590.

In Figure 8a–e, “RO” was mistakenly labelled as “ROC”, and “RO + GS” as “CCG”.

The correct Figure and Legend is below:

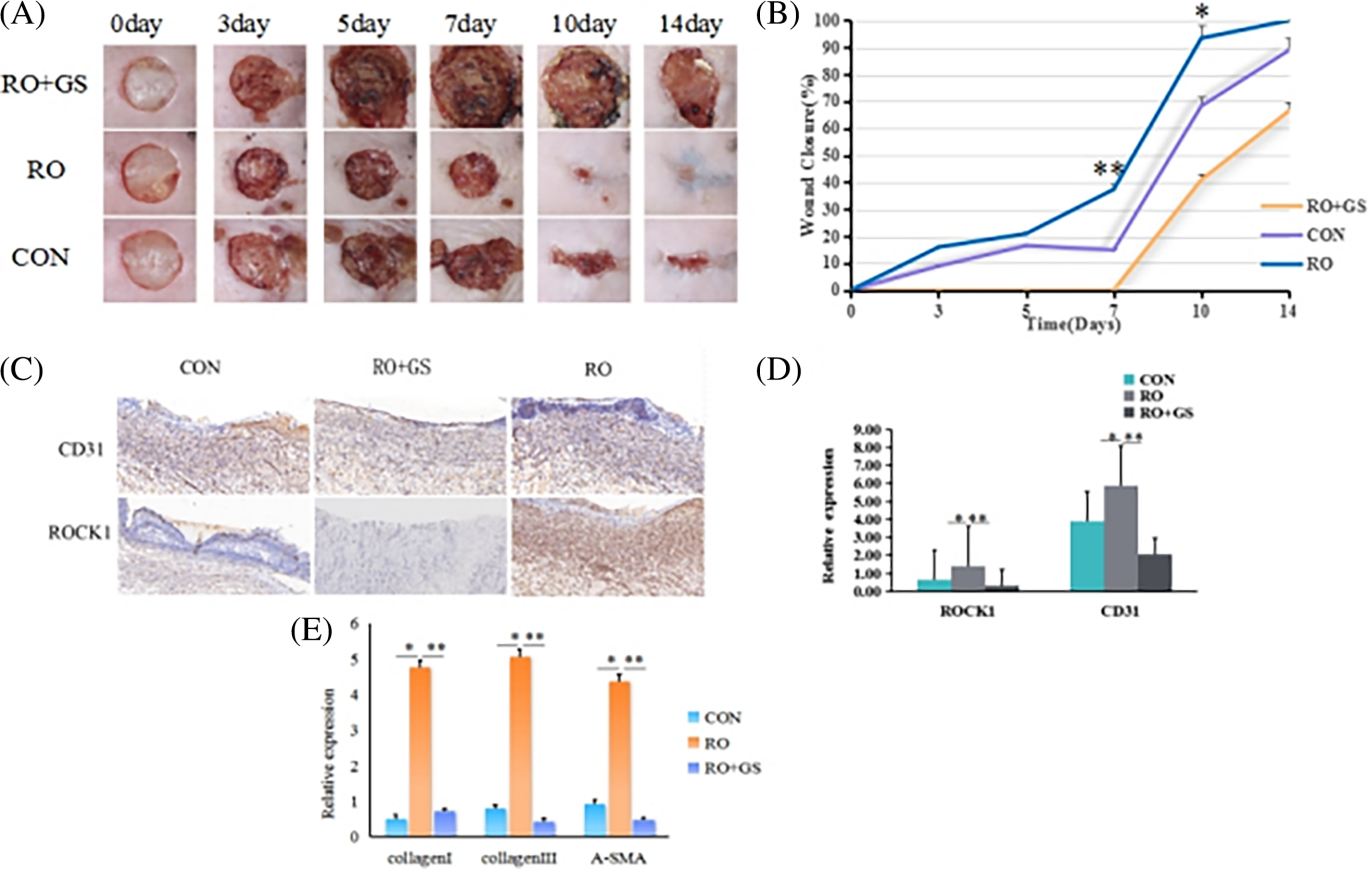

FIGURE 8 Effect of ROCK1 and its inhibitors on wound healing factors (CD31, α‐SMA, Collagen I, Collagen III) in diabetic mice. (A) Representative images of full‐thickness skin defects in rats of CON group, RO group, and RO + GS group immediately, and on days 0, 3, 5, 7, 10 and 14 days postoperatively. (B) Wound closure rate (%). (C) Representative images of (50 ×, scale bar 200 μm) of Immunohistochemistry sections of the CON group, RO group and RO + GS group 14 days postoperatively. (D) Quantitative analysis of CD31 and Rock1 expression. (E) qRT‐PCR analysis detecting α‐SMA, Collagen I, Collagen III gene expression in diabetic tissues of mice. Comparisons were performed using t‐test. *p < 0.05; **p < 0.01. Error bars represent SD (*n* = 5).

We apologize for this error.

